# Olive leaves extract alleviates inflammation and modifies the intrinsic apoptotic signal in the leukemic bone marrow

**DOI:** 10.3389/fimmu.2022.1054186

**Published:** 2023-01-19

**Authors:** Priyatosh Nath, Snehashish Modak, Tamanna Aktar, Sharanya Maiti, Anisha Ghosh, Riddha Singh, Mousumi Debnath, Bhaskar Saha, Debasish Maiti

**Affiliations:** ^1^ Immunology Microbiology Laboratory, Department of Human Physiology, Tripura University, Agartala, Tripura, India; ^2^ Delhi Public School Megacity, Kolkata, West Bengal, India; ^3^ Hariyana Vidyamandir, Kolkata, West Bengal, India; ^4^ Department of Biosciences, Manipal University Jaipur, Jaipur, Rajasthan, India; ^5^ National Centre for Cell Science, Pune, Maharashtra, India

**Keywords:** leukemia, olive leaf, ENU, inflammation, apoptosis

## Abstract

**Introduction:**

Current anti-leukemic chemotherapies with multiple targets suffer from side effects. Synthetic drugs with huge off-target effects are detrimental to leukemic patients. Therefore, natural plant-based products are being increasingly tested for new anti-leukemic therapy with fewer or no side effects. Herein, we report the effect of ethanolic olive leaves extract (EOLE) on the K562 cell line and on the bone marrow (BM) of N-ethyl-N-nitrosourea (ENU)-induced leukemic mice.

**Methods:**

Using standard methodologies, we assessed viability, chromatin condensation, and induction of apoptosis in EOLE-treated K562 cells *in-vitro*. The anti-leukemic activity of EOLE was assayed by measuring ROS, levels of various cytokines, expression of iNOS and COX-2 gene, and changes in the level of important apoptosis regulatory and cell signaling proteins *in-vivo*.

**Result:**

K562 cells underwent apoptotic induction after exposure to EOLE. In the BM of leukemic mice, EOLE therapy decreased the number of blast cells, ROS generation, and expression of NF-κB and ERK1/2. IL-6, IL-1β, TNF-α, iNOS, and COX-2 were among the inflammatory molecules that were down-regulated by EOLE therapy. Additionally, it decreased the expression of anti-apoptotic proteins BCL2A1, BCL-xL, and MCL-1 in the BM of leukemic mice.

**Discussion:**

Chronic inflammation and anomalous apoptotic mechanism both critically contribute to the malignant transformation of cells. Inflammation in the tumor microenvironment promotes the growth, survival, and migration of cancer cells, accelerating the disease. The current investigation showed that EOLE treatment reduces inflammation and alters the expression of apoptosis regulatory protein in the BM of leukemic mice, which may halt the progression of the disease.

## 1 Introduction

Leukemia, a malignant blood disorder, is characterized by an abnormal hematopoiesis and an accumulation of partially differentiated leukemic blasts in the bone marrow (BM). Entry of these blasts into the circulation alters the composition of normal blood and impairs its functions. The leukemic blasts infiltrate and gradually incapacitate the liver, the kidneys, the lungs, the spleen, and the lymph nodes. Leukemia occurs in individuals of all age groups. Relapse in critical patients and resistance to chemotherapy cause severe hematological complications and therapeutic incompatibility specifically after a chemotherapy eventuating in death of the leukemia patients (1). The leukemic cells and other cells in the BM microenvironment secrete several soluble factors in a dysregulated manner, altering cell signalling and aiding the leukemic cells’ survival and resistance to chemotherapy. The leukemic cells become drug resistant by several mechanisms, including inactivation of drug, inhibition of apoptosis, increased drug efflux, decreased drug uptake, altered drug metabolism, enhanced DNA repair and so on (2). The aged leukemia patients cannot sustain intensive chemotherapy due to severe toxicity, or a BM transplant leads to high mortality (3). Overcoming these complications requires the development of therapeutics that will be less toxic, selectively target malignant cells, and strengthen the natural defence mechanism of the individual.

Two important abnormal phenotypes prevalent in leukemia are BM inflammation and apoptotic resistance of malignantly transformed cells (4, 5). The very root of these anomalies lies in the abnormal activation of various signaling molecules, including the nuclear factor-kappa B (NF-κB) and excessive release of various inflammatory cytokines (6, 7). Dysregulation of NF-κB is strongly associated with malignant transformation. It mediates inflammation, accelerates cell proliferation, and makes the cancer cells resistant to apoptosis. It stimulates angiogenesis and promotes metastasis of tumor cells. Constitutive NF-κB activation is commonly seen in Acute Myeloid Leukemia (AML) patients and experimental animal models of AML (7, 8). NF-κB also regulates apoptosis by directly regulating the expression of anti-apoptotic B-cell lymphoma 2-related proteins A1 (BCL2A1) in the murine hematopoietic system (9). Increased anti-apoptotic BCL2A1 and B-cell lymphoma-extra-large (BCL-xL) expression in the mouse hematopoietic system makes the hematopoietic stem and progenitor cells resistant to apoptosis, often leading to hematopoietic transformation and development of leukemia (10, 11). NF-κB is thus an important target for therapeutic intervention in leukemia.

The olive fruit and leaves contain many biologically active polyphenol compounds, including main constituents’ oleuropein and hydroxytyrosol. Both oleuropein and hydroxytyrosol are chemopreventive and kill cancer cells by multiple mechanisms (12, 13). The crude olive extract and the olive polyphenols tested in many tumor cell lines and animal tumor models have shown substantial therapeutic benefits (14). The olive leaf extracts induce apoptosis in K-562 leukemia cells and trigger their differentiation into monocyte-macrophage lineage (15). The EOLE potentiates the antioxidant enzyme functions through up-regulation of nuclear factor erythroid 2-related factor 2 (Nrf2) *in vivo* (16). The EOLE treatment also lowers NF-κB expression and the concentration of inflammatory cytokines in inflamed lungs. This observation is consistent with the available literature describing the pharmacologic properties of crude olive leaf extract, or olive polyphenols, which led us to choose EOLE for the current study. Before finalizing this research design, a pilot study with EOLE was conducted on N-ethyl-N-nitrosourea (ENU) induced leukemic mice substantially reduced the total leukocyte and blast count in the peripheral circulation. This study investigated the anti-leukemic effects of EOLE with reference to its anti-inflammatory and cytotoxic activities.

## 2 Materials and methods

### 2.1 Preparation of plant extract and phytochemical profiling

The fresh leaves of arbequina olive (*Olea europaea*) were provided by the Rajasthan Olive Cultivation Limited (ROCL), Bassi, Rajasthan, India. The plant was authenticated by the plant taxonomist at the Department of Botany, University of Rajasthan. A herbarium of the plant was preserved along the voucher accession number RUBL211669, dated 06/03/2018. The ethanolic extract of the Olive leaves was prepared according to the previously described method (16). The dried extract was kept at -20°C to prevent degradation.

The chemical formulation of EOLE was determined with liquid chromatography-electrospray ionization tandem mass spectrometry (LC-ESI MS/MS) (Agilent Technologies, Palo Alto, CA, USA) (16). The LC-ESI MS/MS analysis identified around 23 different phytochemicals in EOLE; of which, hydroxytyrosol, oleuropein, and apigenin compose significant fractions (16).

### 2.2 Cell culture

Human chronic myelogenous leukemia K562 cell was procured from the National Centre for Cell Science (NCCS), Pune, Maharashtra, India, and was expanded in our laboratory. Cells were propagated with fetal bovine serum (FBS) supplemented RPMI 1640 medium (Gibco) inside a humidified cell culture incubator at 37°C temperature and 5% CO_2_. On reaching about 80% confluence, the culture was split for use in various experiments *in-vitro (* 17).

### 2.3 Cytotoxicity assay

The cytotoxicity of EOLE on K562 cells and naïve mouse splenocytes was investigated by 3-(4,5-Dimethylthiazol-2-yl)-2,5-diphenyltetrazolium bromide (MTT) (Sigma Aldrich) assay in a 96-well plate with slight modification of a standard protocol (18). To each well of 96 well plates 0.1 ml cell suspensions with a cell density of 2×10^5^ cells/ml of RPMI media were seeded and maintained under culture condition. The treated groups of cells were given EOLE (dissolved in cell culture grade dimethyl sulfoxide (DMSO) and successively in RPMI-1640 reducing the final concentration of DMSO to 0.2% only) at the concentrations of 5, 10, 20, 50, and 100 µg/ml of media, whereas the control cells were given 0.2% cell culture grade dimethyl sulfoxide (DMSO). After incubation for 24, 48, and 72 hours the cells were labelled with MTT solution at final concentration of 5mg/ml and incubated for 2-4 hours at 37°C. The resultant formazan crystal formed in control and treated wells was dissolved by the addition of DMSO. The cytotoxic effect of EOLE was calculated as: (%) viability = (absorbance of test-absorbance of blank)/(absorbance of control-absorbance of blank) × 100 (18), [where, the test represents the K562 cells that received EOLE treatment; control represents the untreated K562 cells; and the blank represents only media plus MTT solution and DMSO].

### 2.4 Cell viability assay

The viability of EOLE treated cells was measured by trypan blue dye exclusion assay with minor modification of a standard protocol. The K562 cells were treated with EOLE at 50 and 100µg/ml, incubated for 24 hours and preceded with trypan blue staining. The numbers of live and dead cells were counted under bright-field microscope using a Neubauer haemocytometer. The calculation was done using the formula:

Viable cells (%) = (1 – number of blue or dead cells/total number of cells) x 100 (19).

### 2.5 The AO/EtBr and DAPI staining assay

The Acridine orange/Ethidium bromide (AO/EtBr) and 4′, 6-diamidino-2-phenylindole (DAPI) staining were carried out to study the EOLE-induced cell death and chromatin condensation in K562 cells following standard protocols (20, 21). The calculation was done using the formulae:


Non-apoptotic cells(%)=(1-No. of yellow or orange cells/total no. of cells)×100

Nuclear fragmented cells (%)=(1 – No. of bright blue cells/total no. of cells)×100

### 2.6 DNA ladder assay

The nuclear DNA damage by EOLE was studied using a DNA ladder assay (22). In brief, the DNA from the control and treated K562 cells were electrophoretically separated in 1.5% agarose gel. The DNA bands were visualized under ultraviolet (UV) illumination in a Bio-Rad ChemiDoc™ MP imaging system.

### 2.7 Western blot analysis of apoptosis in K562 cells

Apoptosis induction in EOLE-treated K562 cells was investigated by western blotting (23). An equal amount of protein from the EOLE treated and control K562 cells were separated in SDS-PAGE, transferred to a nitrocellulose membrane, and processed to detect the protein of interest. Primary and secondary antibodies against the target proteins- GAPDH, BCL-2, BCL-xL, BCL2A1, MCL-1, BAX, PUMA, Cytochrome-C, NF-κB, and ERK-1/2 were procured from Abcam (United Kingdom).

### 2.8 Development of leukemia in mice

The animal experiments were performed with the approval from the institutional animal ethics committee (IAEC), reference no. TU/IAEC/2018/XVII/II dated 18th Dec 2018. The healthy BALB/c mice, of both sexes, same age and body weight, were housed at the institutional animal house, maintained under a standard living condition of 25°C ± 2°C temperature, 45% ± 10% relative humidity, 12h light/dark cycle and food and water *ad libitum*. Leukemia in mice was induced by ENU (24). The development of leukemia was confirmed by blasts in the peripheral blood (24). As per our study, 46% mice developed leukemia within 20 weeks after ENU injection.

### 2.9 Exclusion and inclusion criteria for leukemic mice

The primary criteria for exclusion and inclusion of mice into the experiment were the status of leukemia. The mice showing more than 20% myeloid or lymphoid blast in the peripheral blood after five months of ENU injection (25) were considered leukemic. The leukemic mice with excessive weakness or very low body weight were excluded from the studies.

### 2.10 Mice groups and treatment schedule for *in-vivo* studies

Four different groups of mice were taken for this study (n=6 in each group) (26). Treatment was given by oral gavage for 1 month. Details of animal groups and treatment are: Group-1: Normal control- given sterile water; Group-2: Leukemia control- given sterile water; Group-3: Normal + EOLE- given EOLE in water at 200mg/kg body weight; and Group-4: Leukemia + EOLE- given EOLE dissolved in water at 200mg/kg body weight.

A toxicity study (unpublished) of EOLE was performed in mice before choosing the dose for the *in-vivo* treatment. In brief, the EOLE was orally administered in different groups of mice at the doses of 50, 100, 200, 500, and 1000mg/kg body weight daily for 28 days. During this period the animals were observed for changes in food and water intake, body weight and abnormal symptoms, physical weakness, and death. Notably, the mice group given EOLE at 1000mg/kg body weight developed diarrhoea within 3 to 4 days of continued oral administration. However, no such symptoms were seen in the mice groups given EOLE at 50, 100, and 200mg/kg body weight. The food and water intake, body weight, and physical status in these groups of animals were found similar to the control mice. Therefore, EOLE at 200mg/kg body weight was chosen for *in-vivo* treatment.

### 2.11 Animal sacrifice and sample collection

On completion of treatment, all the mice under the experiment were sacrificed following the IAEC guidelines. Mice were allowed to inhale chloroform and euthanized by cervical dislocation. The mice assorted in groups were then dissected in aseptic conditions to collect the organs, spleen, and hind limbs to isolate BM from femur bones. Blood was collected before euthanasia by puncturing the retro-orbital sinus with a sterile capillary tube.

### 2.12 Complete blood count and morphological study

The complete blood count (CBC) of EDTA containing peripheral blood samples was performed in the Trivitron (Celenium-19) digital blood analyzer system (23). The blast cell counting was performed manually under the microscope. Morphology of blood cells was examined conventionally. In brief, a thin smear of blood was prepared and stained using a standard protocol (27). The observation was carried out under 40X and 100X objectives in bright-field microscopy, Leica DM4000B LED fluorescence microscope.

### 2.13 Serum lactate dehydrogenase activity

The lactate dehydrogenase activity in serum was measured with little modification of the protocol of Simaga et al., 2008 (28). The activity was measured spectrophotometrically at room temperature for 3 min by recording decrease in absorbance of NADH at 340 nm in UV 1900 Shimadzu UV visible double beam spectrophotometer.

### 2.14 Bone marrow smear preparation and morphological study

The femoral marrow cells were isolated (29). A thin smear of the BM cells was drawn on a grease-free glass slide, stained with Leishman’s stain (27) and was observed under a Leica DM4000B LED fluorescence microscope.

### 2.15 Determination of inflammatory and hematopoietic cytokines in the BM

The indicated cytokines in the BM was measured using the method described by Pino et al., 2010 (30). The supernatants of PBS suspended BM samples were assayed for the indicated cytokines such as IL-1α, IL-1β, TNF-α, TGF-β, VEGF, SCF, G-CSF, GM-CSF, IL-3, and IL-6 by respective cytokine ELISA kits following the manufacturer’s protocol (PeproTech, USA and ImmunoTools GmbH, Germany).

### 2.16 Immunoblots for apoptosis-related and signaling proteins in the BM

The total protein was isolated from the BM cells, resolved on polyacrylamide gels, transferred onto PVDF membrane and probed with the respective primary and secondary antibodies (Abcam, United Kingdom) (31).

### 2.17 Measurement of ROS production in bone marrow

ROS production in BM was measured following a standard protocol (32). This method used 2-,7-dichlorofluorescein diacetate (DCFDA) as a fluorescence probe. The fluorescence intensity of the samples was taken at λ_excitation_ = 488nm and λ_emission_ = 530nm in Synergy H1 Hybrid Reader (BioTek Instruments, Inc., Winooski, USA).

### 2.18 Expression studies of iNOS and COX-2 in bone marrow by real-time PCR

RNA from the BM cells was extracted using Trizol reagent (Ambion by Life Technologies, #15596018). The RNA in equal amount was taken from each sample to synthesize cDNA using the BIO-RAD iScript ™ cDNA synthesis kit (#170-8891). The gene expression was studied in an Applied Biosystem’s Step-One Plus real-time PCR system following a standard protocol (16). The sequence of the PCR primer used are: GAPDH Forward: 5′-CAC CAC CCT GTT GCT GTA GCC-3′; GAPDH Reverse: 5′- ACC ACA GTC CAT GCC ATC AC-3′; iNOS Forward: 5’- GCC ACC AAC AAT GGC AAC A-3’; iNOS Reverse: 5’- CGT ACC GGA TGA GCT GTG AAT T-3’; COX-2 Forward: 5’- GAA GAT TCC CTC CGG TGT TT-3’; COX-2 Reverse: 5’- CCC TTC TCA CTG GCT TAT GTA G-3’

### 2.19 Immunohistochemistry of spleen

Splenic sections were stained for immunohistochemistry and detection of leukemic blast using a standard protocol (10). In brief, thin sections of spleen were deparaffinised and hydrated before incubating in hydrogen peroxide (H2O2) solution to block endogenous peroxidase activity. The sections were blocked with bovine serum albumin (BSA) solution and incubated with anti-mouse rabbit polyclonal antibody to BCL2A1/GRS and MCL-1 antibody for overnight. On the next day, the sections were washed with PBS and incubated with goat anti-rabbit IgG H&L (HRP) (cat. ab97051). Finally, the sections were stained with diaminobenzidine (DAB) and followed by counterstaining with hematoxylin. The sections were then examined and photographed under a Leica DM4000B LED microscope.

### 2.20 Preparation and isolation of mouse peritoneal macrophage

The peritoneal macrophage from the BALB/c mice was isolated with a slight modification of a standard protocol (33). The macrophage cells were maintained with the serum-supplemented DMEM/F12 medium.

### 2.21 Macrophage inflammation assay

The effect of EOLE on macrophages- Macrophage + PBS (Normal control); Macrophage + LPS (Inflammation control); Macrophage + LPS &PBS (Treatment-1); Macrophage + LPS and EOLE at 50µg/ml (Treatment-2); Macrophage + LPS and EOLE at 100µg/ml (Treatment-3)- was assayed *in-vitro* (34). Lipopolysaccharide (LPS) was added to the macrophages at the final concentration of 100ng/ml. The LPS stimulates the macrophage to release TNF-α and IL-1β which were studied in the presence and absence of EOLE.

### 2.22 Statistical analysis

The statistical analysis was performed in GraphPad InStat 3 software. Statistical significance was tested by one-way ANOVA followed by Tukey’s multiple comparison tests between the control and treated groups. The p-value p<0.05 was considered statistically significant.

## 3 Results

### 3.1 EOLE is cytotoxic and induces apoptosis to the K562 cells

**Figure 1:**
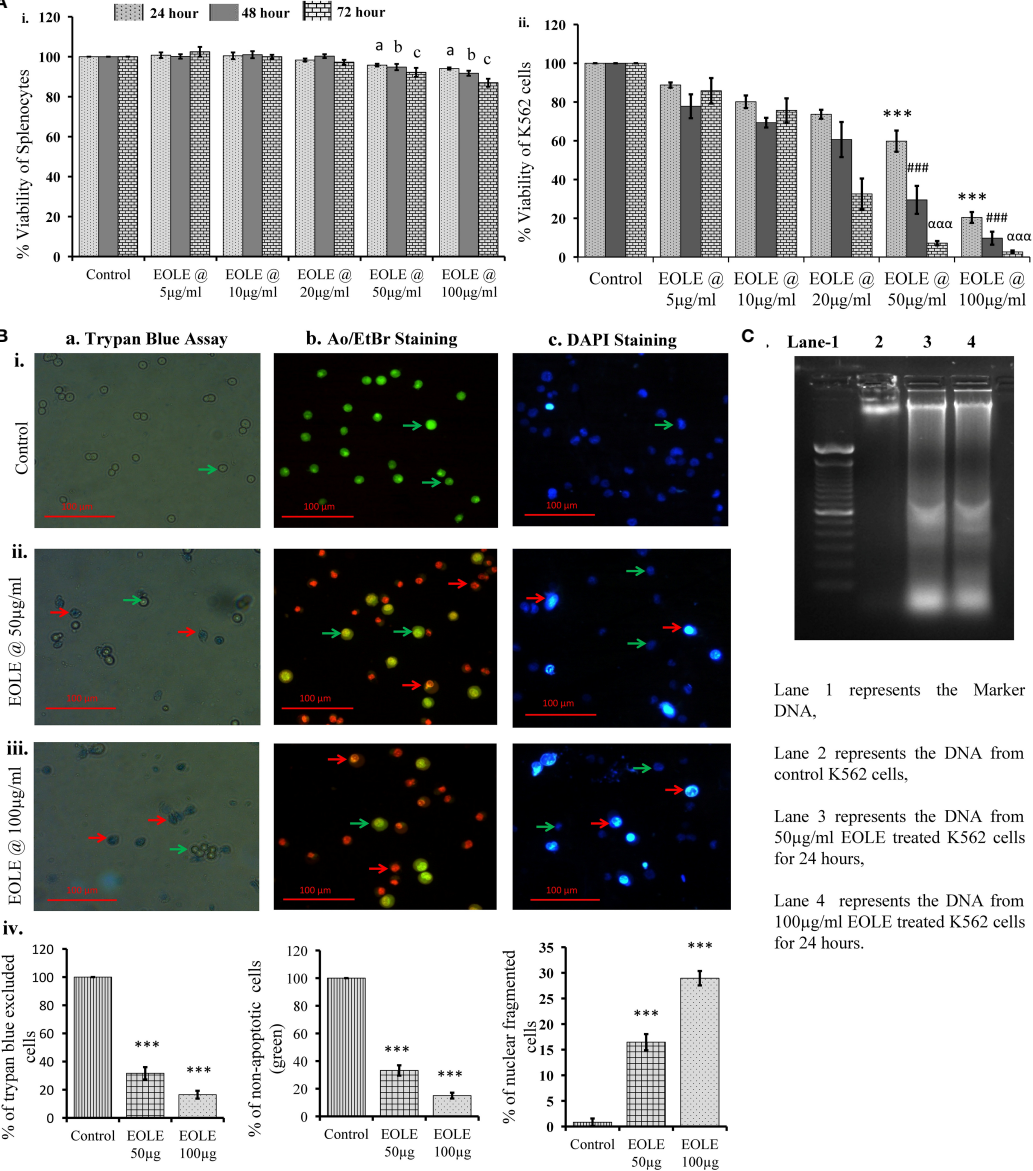
Figures showing the cytotoxic activity of EOLE: **(A)** Viability percentage of control and EOLE-treated K562 and splenocytes evaluated through MTT assay, where (i) Viability of mouse splenocytes, (ii) Viability of K562 cells; values are expressed as mean ± SD of 3 observations. The statistical significance was tested using one-way ANOVA following Tukey’s multiple comparison tests by comparing the values as, control *vs*. treatments separately for 24 hours, 48 hours, and 72 hours. P value, p < 0.05 was considered as statistically significant. When compared between control *vs*. treatment for 24 hours the level of significance is denoted as, a = not significant when p > 0.05; * when p < 0.05; ** when p < 0.01; and *** when p < 0.001. When compared between control *vs*. treatment for 48 hours the level of significance is denoted as, b = not significant when p > 0.05; **#** when p < 0.05; **##** when p < 0.01; and **###** when p < 0.001. When compared between control *vs*. treatment for 72 hours the level of significance is denoted as, c = not significant when p > 0.05; **α** when p < 0.05; **αα** when p < 0.01; and **ααα** when p < 0.001; **(B)** Result of the trypan blue dye exclusion assay, AO/EtBr staining, and DAPI staining, where (a) Trypan blue assay, (b) AO/EtBr staining, (c) DAPI staining: (i) Representative images of control group, (ii) Representative images of EOLE-treated (50 µg/ml) group, (iii) Representative images of EOLE-treated (100 µg/ml) group, (iv) Bar diagrams showing results of staining assay. Values are expressed as mean ± SD of 3 different observations. The statistical significance was tested using one-way ANOVA followed by Tukey’s multiple comparison tests by comparing the values as, control *vs*. treatments. P-value, p < 0.05 was considered as statistically significant and denoted as, a = not significant when p > 0.05; * when p < 0.05; ** when p < 0.01; and *** when p < 0.001; **(C)** The representative image of the DNA ladder assay.

MTT assay showed that EOLE (up to 100µg/ml) had significant toxicity against K562 cells but not against naïve mouse splenocytes. At the given dose of EOLE 50 µg/ml, the viability percentage of K562 cells reduced significantly to 59.78 ± 5.46 (p<0.001), 29.48 ± 7.2 (p<0.001), and 7.16 ± 1.1 (p<0.001), respectively, after 24, 48 and 72 hours of incubation. The viability percentage of K562 cells further reduced to 20.42 ± 2.79 (p< 0.001), 9.72 ± 3.36 (p< 0.001), and 2.64 ± 0.71 (p< 0.001), respectively, for 24, 48, and 72 hours at the EOLE dose of 100 µg/ml (**Figure 1A** i & ii). The trypan blue dye exclusion experiment confirmed the above data (**Figure 1B** a. i to iv). The control and treated K562 cells were also stained with the AO/EtBr and DAPI and observed under fluorescence microscope to visualize the apoptotic features like membrane blebbing and chromatin condensation. AO/EtBr gives distinctive characteristic features to normal and apoptotic cells. The normal cell fluoresced green; however, the early apoptotic cell showed bright yellow areas in the nucleus. Concurrently, the late apoptotic cell took up EtBr due to loss of membrane integrity and fluoresced orange (**Figure 1B** b. i to iv). The percentage of non-apoptotic K562 cells presented along a bar diagram showed a significantly (*p<*0.001) less non-apoptotic K562 cells in EOLE treated groups compared to control group (**Figure 1B** b. iv). The DAPI staining of K562 cells reflects the changes in the nucleus. The control K562 cells showed round to oval-shaped nuclei with diffuse blue staining, whereas many cells in EOLE treated groups fluoresce bright blue indicating chromatin condensation and nuclear fragmentation (**Figure 1B** c. i to iv). The number of chromatin condensed cell was found significantly higher in 100µg/ml EOLE treated group (Fig- 1Bc. iv). DNA fragmentation in K562 cells was also assayed by DNA ladder assay. The DNA from the control K562 cells produced a thick band in the agarose gel close to the loading well, whereas the DNA from EOLE-treated K562 cells produced a smear due to DNA fragmentation by endonucleases (**Figure 1C**).

### 3.2 EOLE induces the expression of apoptotic proteins in K562 cells

**Figure 2:**
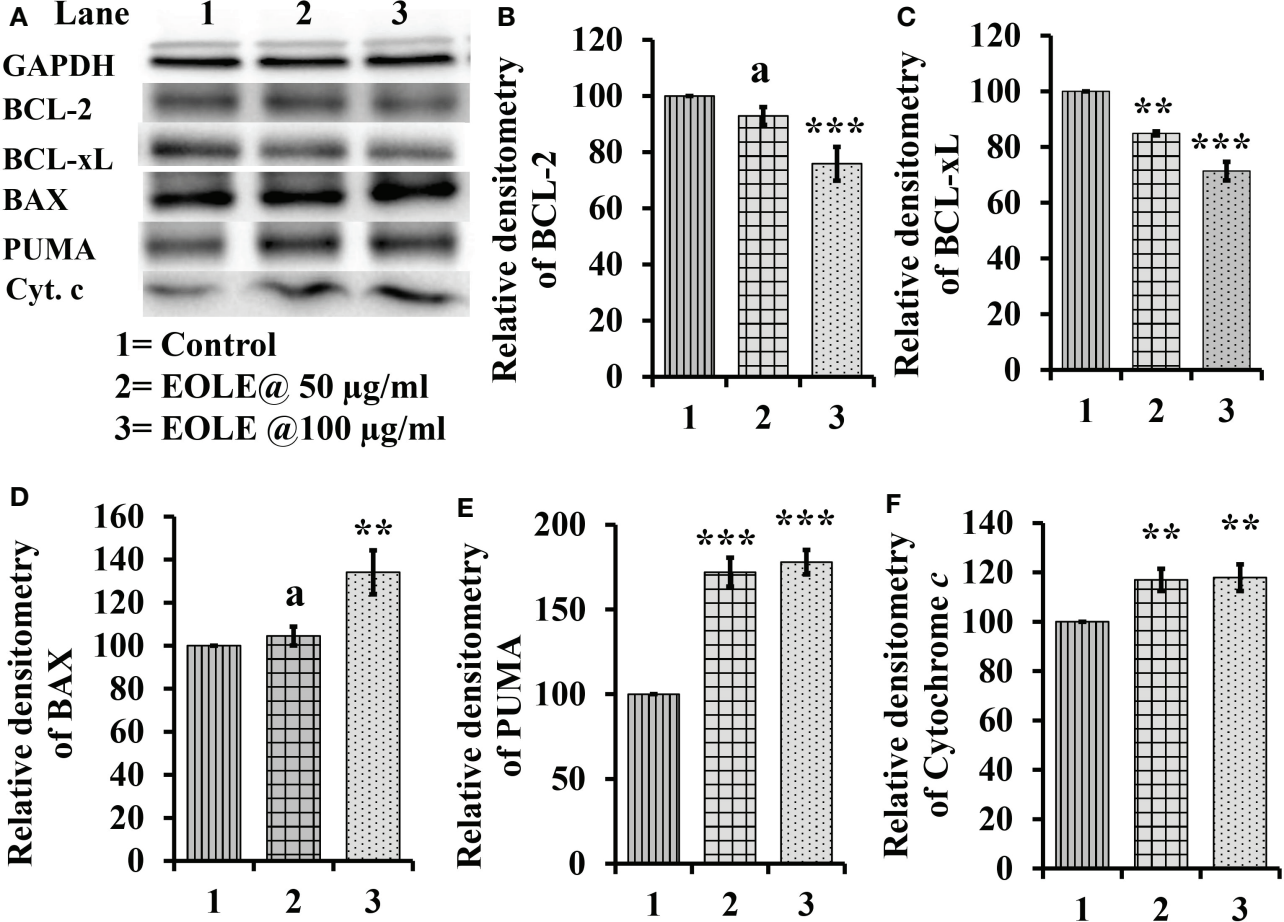
Figure showing the expression of many apoptotic pathway proteins in control and EOLE treated K562 cells, where **(A)** Images of protein bands, **(B)** Densitometry plot for BCL-2, **(C)** Densitometry plot for BCL-xL, **(D)** Densitometry plot for BAX, **(E)** Densitometry plot for PUMA, **(F)** Densitometry plot for cytochrome *c* Values are expressed as mean ± SD of 3 observations. The statistical significance was tested using one-way ANOVA following Tukey’s multiple comparison tests by comparing the values as, control *vs*. treatments. P-value, p<0.05 was considered as statistically significant and denoted as a= not significant when p > 0.05; * when p < 0.05; ** when p < 0.01; and *** when p < 0.001.

The cytoplasm of an apoptotic cell always possesses more pro-apoptotic proteins and fewer anti-apoptotic proteins. Therefore, the changes in BCL-2, BCL-xL, BAX, PUMA, and cytochrome c proteins in the control and treated K562 cells were studied by western blotting (**Figure 2**). The densitometry analysis of protein bands showed a significant reduction in anti-apoptotic proteins BCL-2 (*p<*0.001) (**Figure 2B**), BCL-xL (*p<*0.001) (**Figure 2C**); and an increase in pro-apoptotic proteins BAX (*p<*0.01) (**Figure 2D**), and PUMA (*p<*0.001) (**Figure 2E**) in treated K562 cells at the concentration of 50µg and 100 µg EOLE/ml of media. The western blot study also showed significantly more (p<0.01) cytochrome c in the treated K562 cells than the control due to the increased release of cytochrome c from the mitochondria of EOLE-induced apoptotic cells (**Figure 2F**).

### 3.3 EOLE reduced leukemic blast cell in blood and bone marrow in mice model

**Figure 3:**
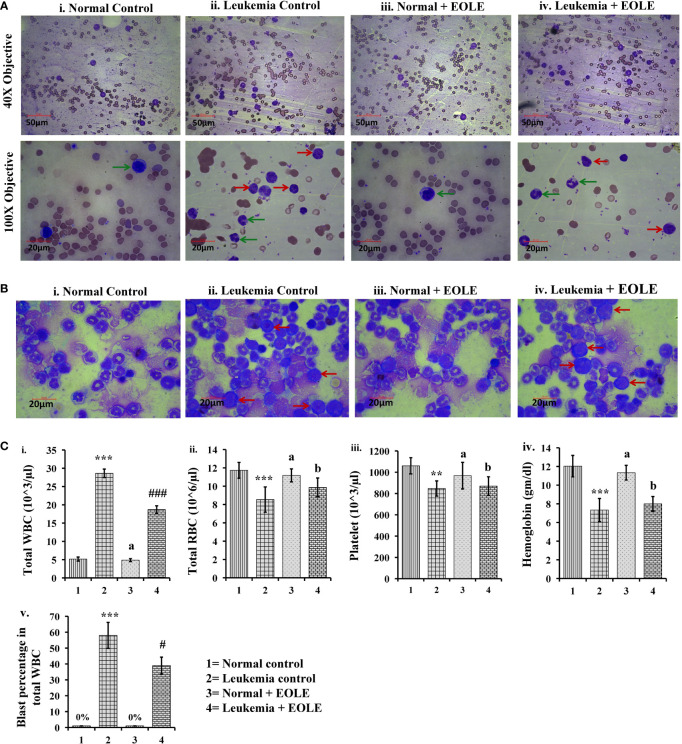
Figures showing the morphology of blood bone marrow and various hematologic parameters: **(A)** Representative images of blood smears at different magnifications, where (i) Normal control, (ii) Leukemia control, (iii) Normal + EOLE, (iv) Leukemia+ EOLE. In these figures, the red arrow indicates leukemic blast cells, and the green arrow indicates normal WBCs; **(B)** Representative images of bone marrow, where (i) Normal control, (ii) Leukemia control, (iii). Normal + EOLE, (iv). Leukemia + EOLE. In these figures, the red arrow indicates leukemic blast cells; **(C)** Bar diagrams representing various haematological parameters, where (i) WBC count, (ii) RBC count, (iii) Platelet count, (iv) Hemoglobin concentration, (v) Blast cell count (percentage of total WBC). Values are expressed as mean ± SD of 3 different observations. The statistical significance was tested using one-way ANOVA following Tukey’s multiple comparison tests by comparing the values as, Group-1 *vs*. Group-2, 3; Group-2 *vs*. Group- 4. P value, p < 0.05 was considered as statistically significant. When compared with Group-1 the level of significance was denoted as, a = not significant when p > 0.05; * when p < 0.05; ** when p < 0.01; and *** when p < 0.001. When compared with Group-2 the level of significance was denoted as, b = not significant when p > 0.05; # when p < 0.05; ## when p < 0.01; and ### when p < 0.001.

Leukemia was developed in mice within 20 weeks of ENU challenge. In leukemic mice, the leucocyte count increased several folds. A significant part of these leucocyte comprised leukemic blasts characterized by a large nucleus and scant cytoplasm. These blasts accumulated in the BM and blood. The microscopic observation of blood and BM smears revealed a significantly (*p<*0.05) lesser blast count in EOLE-treated leukemic mice compared to leukemic control (**Figures 3A, B**). The total count of blood corpuscles and hemoglobin showed a considerable difference among the studied animal groups. The total leukocyte count in the EOLE-treated leukemic group was found significantly (*p<*0.001) lesser compared to leukemic control mice (**Figure 3C**). The RBC count, platelet count, and hemoglobin, which were significantly less (*p<*0.01) in the leukemic control mice, moderately increased in EOLE-treated leukemic mice (**Figure 3C**).

### 3.4 EOLE rescues the body weight and improves LDH activity

**Figure 4:**
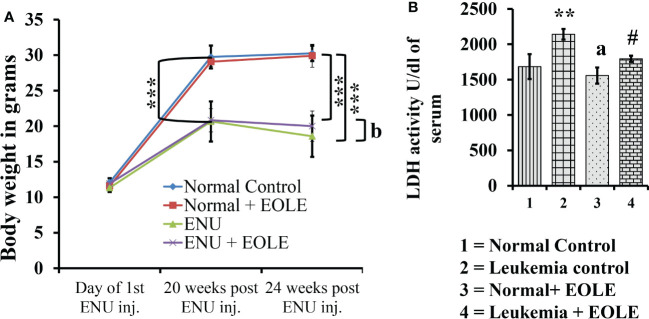
The figures show the effect of EOLE treatment on the health parameter of mice, where **(A)** Effect on body weight. Values are expressed as mean ± SD for 6 mice. **(B)** Lactate dehydrogenase activity (LDH) assay. Values are expressed as mean ± SD of 3 different observations. The statistical significance was tested using one-way ANOVA following Tukey’s multiple comparison tests by comparing the values as, Group-1 *vs*. Group-2, 3; Group-2 *vs*. Group- 4. P-value, p < 0.05 was considered as statistically significant (n = 6).When compared with Group-1 the level of significance was denoted as, a = not significant when p > 0.05; * when p < 0.05; ** when p < 0.01; and *** when p < 0.001. When compared with Group-2, b = not significant when p > 0.05; # when p < 0.05; ## when p < 0.01; and ### when p < 0.001.

The average body weight of mice differs among the studied groups of mice. The experiments started by taking mice of almost the same body weight of 11 to 12 gm; however, after the 20^th^ week of ENU administration, the weight of the mice showed a significant difference between the normal and ENU-injected groups. The average weight of ENU-induced leukemic mice was measured significantly (*p<*0.001) less than the average weight of normal mice at 20^th^ and 24^th^ weeks. After 20 weeks the body weight of the leukemic mice showed slight reduction, however when compared the weight loss was found substantially more in leukemic control mice than EOLE-treated group but not statistically significant (*p>*0.05) (**Figure 4A**).

LDH is a prognostic marker of cancer progression. In this study, the serum LDH activity was measured significantly (*p<0.01*) higher in leukemic control group than in the normal control. However, in the EOLE-treated leukemic group, it was measured significantly (*p<*0.05) less than in the leukemic control group (**Figure 4B**).

### 3.5 EOLE reduces the expression of hematopoietic cytokines- SCF, IL-3, IL-6, GCSF and GM-CSF- in the leukemic bone marrow

**Figure 5:**
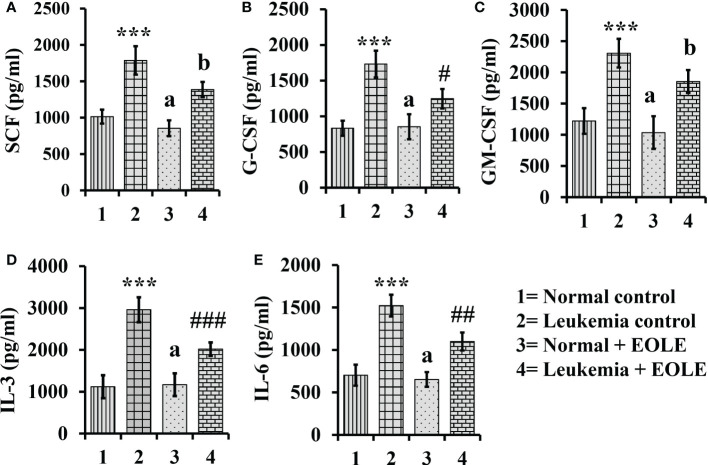
Bar diagrams showing the effect of EOLE treatment in the bone marrow concentrations of various hematopoietic cytokines, where **(A)** SCF, **(B)** G-CSF, **(C)** GM-CSF, **(D)** IL-3, **(E)** IL-6; values are expressed as mean ± SD of 3 different observations. The statistical significance was tested using one-way ANOVA and Tukey’s multiple comparison tests by comparing the values as, Group-1 *vs*. Group-2, 3; Group-2 *vs*. Group- 4. P value, p < 0.05 was considered as statistically significant. When compared with Group-1 the level of significance was denoted as, a = not significant when p > 0.05; * when p < 0.05; ** when p < 0.01; and *** when p < 0.001. When compared with Group-2, b = not significant when p >0.05; # when p < 0.05; ## when p < 0.01; and ### when p < 0.001.

Hematopoiesis from the hematopoietic stem cells (HSCs) is a complex process tightly regulated by the hematopoietic cytokines in the BM. The level of major hematopoietic cytokines like G-CSF, GM-CSF, SCF, IL-3, and IL-6 measured in the BM showed variation among different animal groups. The result of ELISA revealed a significantly (*p<*0.001) higher level of these cytokines in the BM of leukemic control mice. However, the leukemic mice that received EOLE treatment had significantly less G-CSF (*p<*0.05), IL-3 (*p<*0.001), and IL-6 (*p<*0.01) (**Figure 5**).

### 3.6 EOLE inhibits the inflammation by reducing expression of inflammatory markers

**Figure 6:**
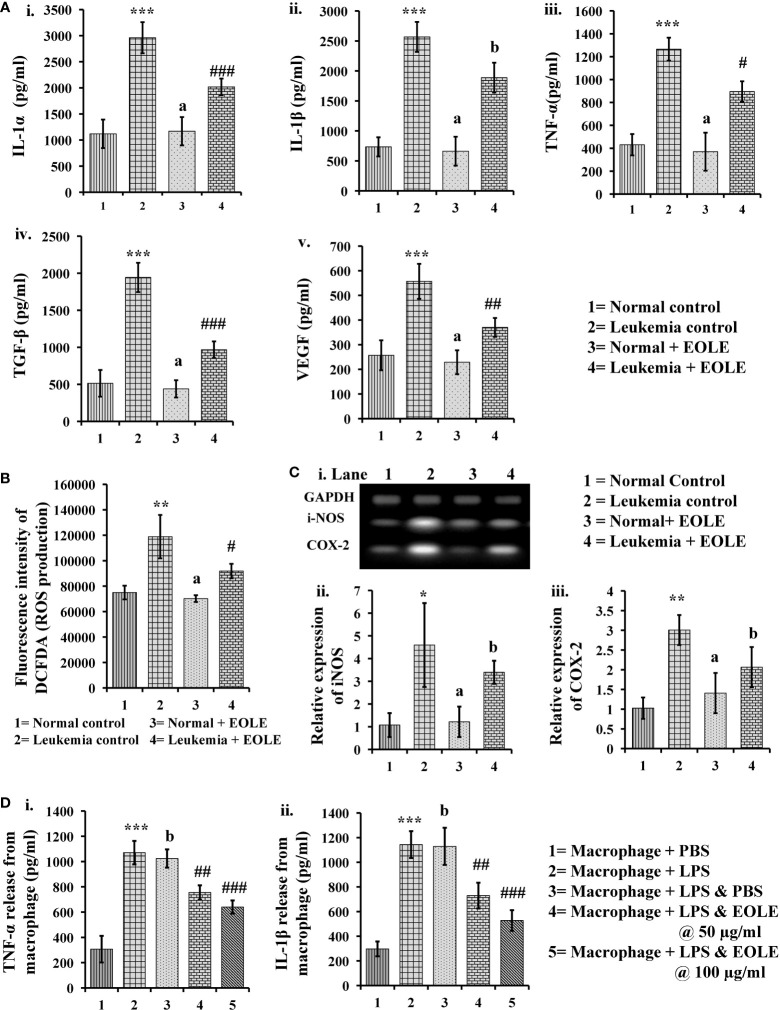
Figures showing the result of EOLE treatment on the expression of various inflammatory substances: **(A)** Bar diagrams showing the concentrations of various inflammatory cytokines in BM, where (i) IL-1α, (ii) IL-1β, (iii) TNF-α, (iv) TGF-β, (v) VEGF; **(B)** ROS production by the BM cells; **(C)** Result of gene expression studies, where (i) Bands of PCR amplified products, (ii) Relative expression of iNOS, (iii) Relative expression of COX-2; values are expressed as mean ± SD of 3 different observations. The statistical significance was tested using one-way ANOVA following Tukey’s multiple comparison tests by comparing the values as, Group-1 *vs*. Group-2, 3; Group-2 *vs*. Group- 4. P value, p < 0.05 was considered as statistically significant. When compared with Group-1 the level of significance was denoted as, a = not significant when p > 0.05; * when p < 0.05; ** when p < 0.01; and *** when p < 0.001. When compared with Group-2, b = not significant when p > 0.05; # when p < 0.05; ## when p < 0.01; and ### when p<0.001; **(D)** Bar diagrams representing the result of macrophage inflammation assay, where (i) TNF-α release, (ii) IL-1β release; values are expressed as mean ± SD of 3 different observations. The statistical significance was analyzed by comparing the values as, Group-1 *vs*. Group-2; Group-2 *vs*. Group- 3, 4, 5. P value, p < 0.05 was considered as statistically significant. When compared with Group-1, a = not significant when p > 0.05; * when p < 0.05; ** when p < 0.01; and *** when p < 0.001. When compared with Group-2, b = not significant when p > 0.05; # when p < 0.05; ## when p < 0.01; and ### when p < 0.001.

Inflammatory cytokines up-regulate in leukemia support the progression of leukemia. In this study, the protein levels of IL-1α, IL-1β, TNF-α, TGF-β, and VEGF in BM were measured significantly (*p*<0.001) higher in the leukemic control group compared to the normal control. However, the level of these cytokines significantly reduced (IL- 1α (p<0.001), TNF-α (p<0.05), TGF-β (p<0.001), and VEGF (p<0.01)) after EOLE treatment (**Figure 6A**). ROS is an essential mediator of inflammation. The ROS level in the BM was found significantly (*p<*0.01) higher in the leukemic control group compared to normal control. EOLE treatment of leukemic mice significantly (*p<*0.05) reduced ROS production (**Figure 6B**). Among the other inflammatory markers, the expression of iNOS and the COX-2 gene was measured in the BM of different groups of mice. A significantly higher expression of iNOS (*p*<0.05) and COX-2 (*p*<0.01) was found in the BM of the leukemic control group compared to the normal control. In the EOLE-treated leukemic group, the expression of iNOS and COX-2 was measured moderately less than in the leukemic control group (**Figure 6C**).

The anti-inflammatory activity of EOLE was further assayed in LPS-induced mouse peritoneal macrophages. LPS stimulates the macrophage to release TNF-α and IL-1β are measured *in-vitro*. The EOLE was found to affect the release of TNF-α and IL-1β from LPS-stimulated macrophages. The culture supernatants assayed through ELISA revealed a significantly (*p<* 0.001) higher TNF-α (**Figure 6D**i) and IL-1β (**Figure 6D**ii) levels. However, the release of TNF-α and IL-1β was reduced significantly (*p<* 0.001) in the presence of EOLE.

### 3.7 EOLE alters intrinsic apoptotic signals in the BM of leukemic mice

**Figure 7:**
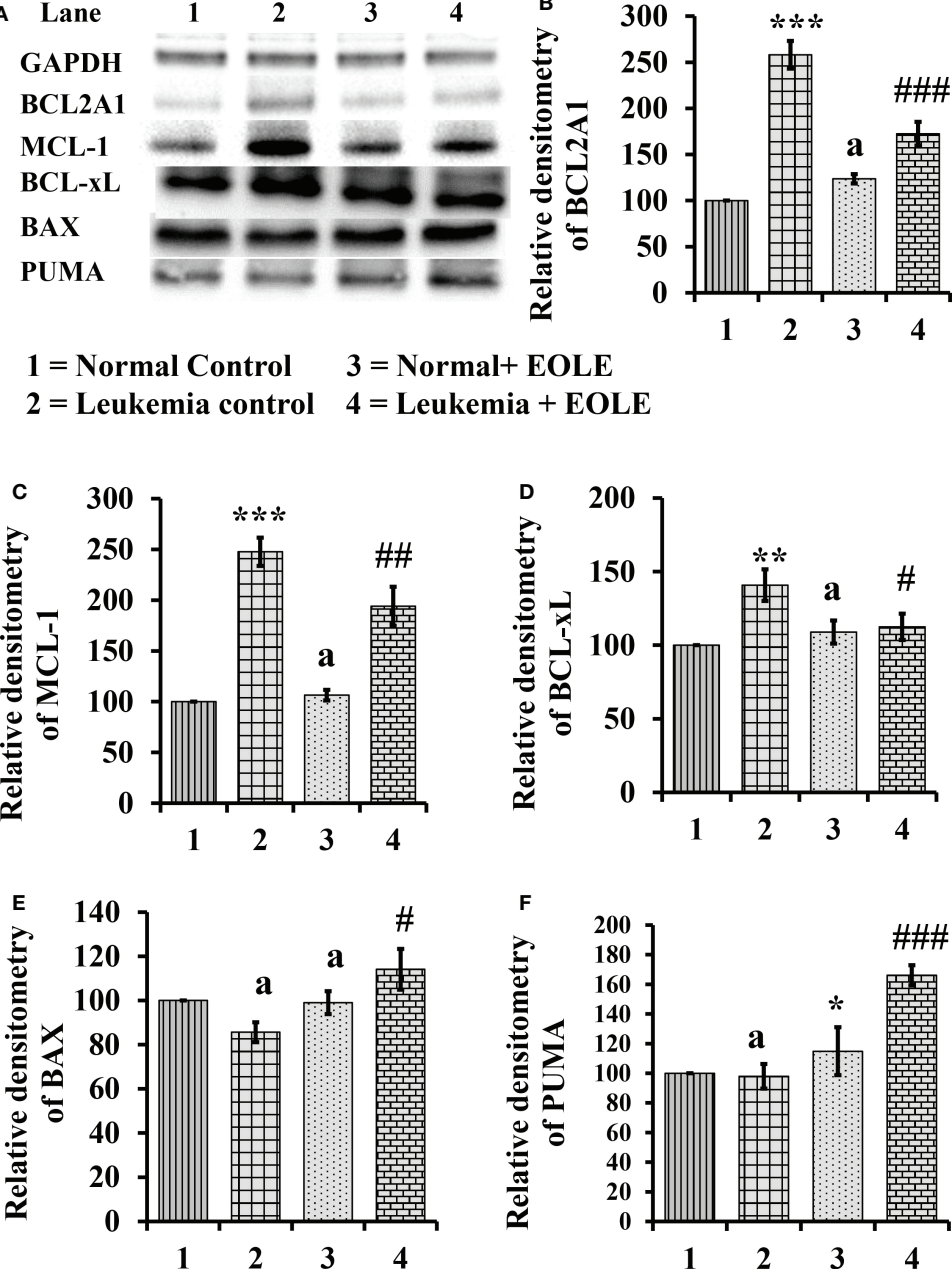
Figure showing the result of western blot analysis for many apoptotic pathway proteins in the bone marrow of mice, where **(A)** Image of protein bands, **(B)** Densitometry plot for BCL2A1, **(C)** Densitometry plot for MCL-1, **(D)** Densitometry plot for BCL-xL, **(E)** Densitometry plot for BAX, **(F)** Densitometry plot for PUMA. Values are expressed as mean ± SD of 3 observations. The statistical significance was tested using one-way ANOVA following Tukey’s multiple comparison tests by comparing the values as, Group-1 *vs*. Group-2, 3; Group-2 *vs*. Group- 4. P-value, p < 0.05 was considered as statistically significant. When compared with Group-1 the level of significance was denoted as, a = not significant when p > 0.05; * when p < 0.05; ** when p < 0.01; and *** when p < 0.001. When compared with Group-2, b = not significant when p > 0.05; # when p < 0.05; ##when p < 0.01; and ###when p < 0.001.

Apoptotic resistance is one of the fundamental properties of cancer cells. In this study, the expression of anti-apoptotic BCL2A1, BCL-xL, and MCL-1 proteins was found significantly higher [(*p<*0.001), (*p<*0.01), and (*p<*0.001), respectively] in the BM of ENU-induced leukemic mice with lesser expression of pro-apoptotic proteins BAX and PUMA than in control mice. However, following EOLE treatment the expression pattern of the pro-apoptotic and anti-apoptotic proteins was reversed significantly (**Figure 7**).

### 3.8 NF-κB and ERK 1/2 were down-regulated by EOLE in leukemic bone marrow

**Figure 8:**
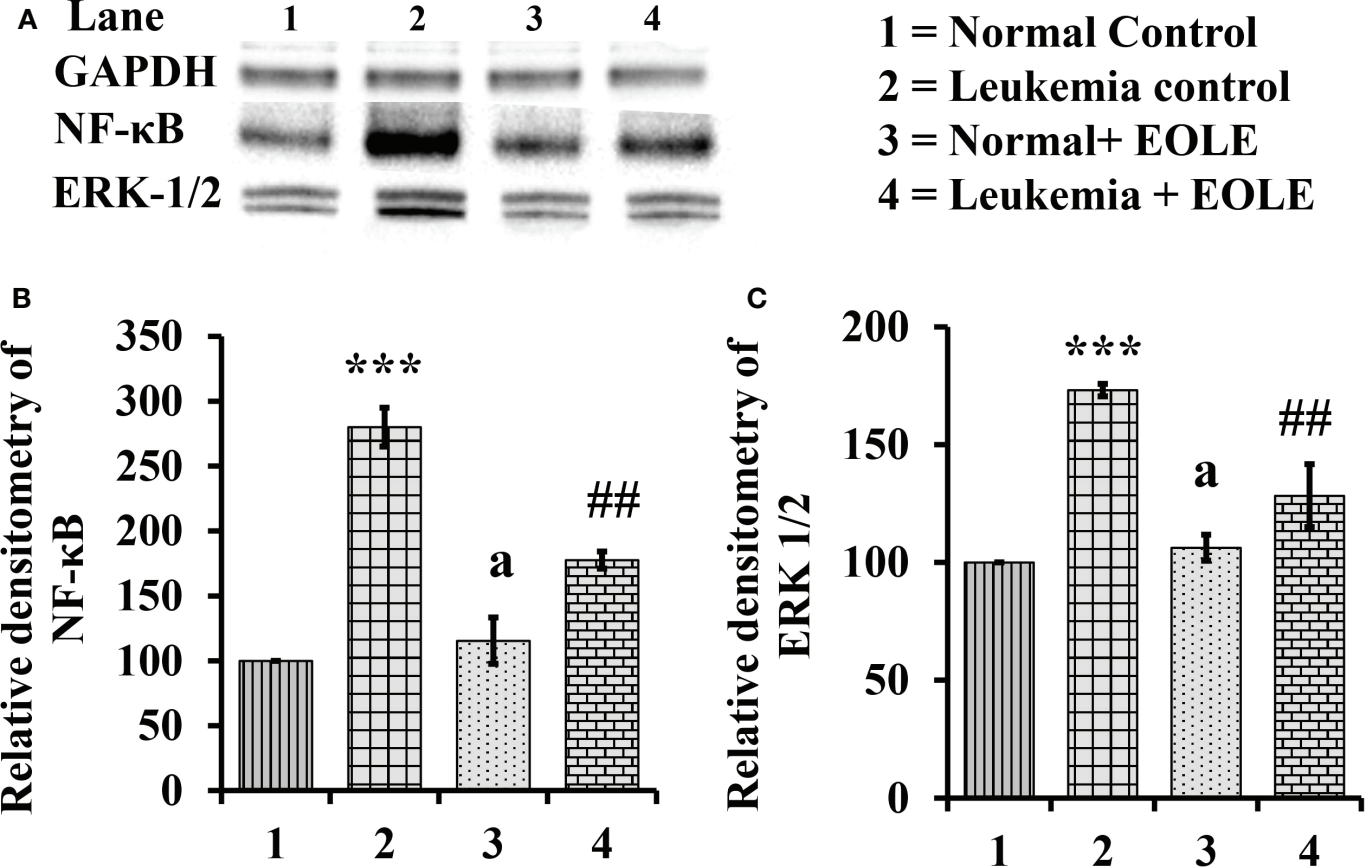
Figure showing the expression of NF-κB and ERK1/2 in the bone marrow of mice, where **(A)** Image of NF-κB and ERK1/2 protein bands, **(B)** Densitometry plot for NF-κB, **(C)** Densitometry plot for ERK1/2; values are expressed as mean ± SD of 3 observations. The statistical significance was tested using one-way ANOVA following Tukey’s multiple comparison tests by comparing the values as, Group-1 *vs*. Group-2, 3; Group-2 *vs*. Group- 4. P-value, p < 0.05 was considered as statistically significant. When compared with Group-1 the level of significance was denoted as, a = not significant when p > 0.05; * when p < 0.05; ** when p < 0.01; and ***when p < 0.001. When compared with Group-2, b = not significant when p > 0.05; # when p < 0.05; ## when p < 0.01; ### when p < 0.001.

The NF-κB in general induces the expression of several genes that play critical role in cell proliferation and apoptosis, so, the expression of NF-κB in the BM from different groups of mice were assayed. The study revealed a significantly (*p<*0.001) increased expression of NF-κB protein in the BM of leukemic mice. However, it was significantly (*p<*0.01) down-regulated in the EOLE-treated leukemic group (**Figures 8A, B**).

Similarly the ERK1/2, that transduce extracellular signals to the interior of cell showed aberrant expression. In this study, the western blot analysis of BM cell protein revealed a significantly (*p<*0.001) increased ERK1/2 in leukemic control group compared to normal counterpart. However, in EOLE treated leukemic group, the expression of the same protein was significantly (*p<*0.01) reduced (**Figures 8A, C**).

### 3.9 EOLE reduced infiltration of leukemic blast in the spleen

**Figure 9:**
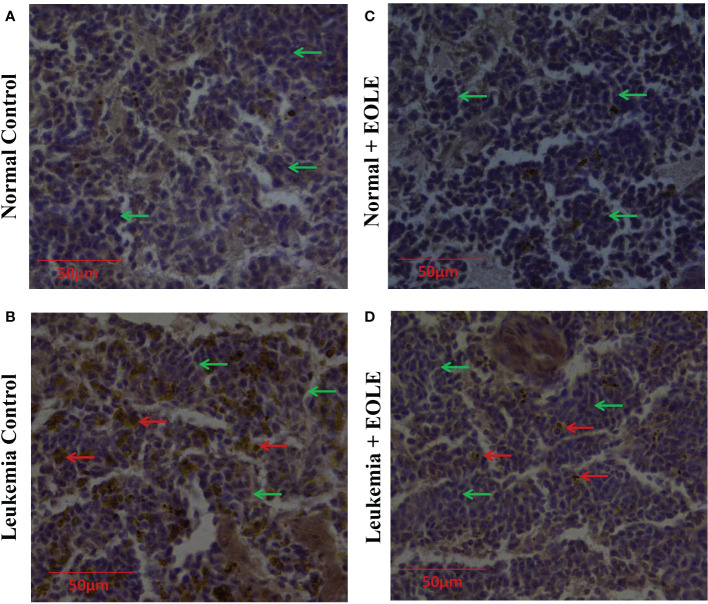
Immunohistochemistry images of splenic sections for BCLA1 protein. **(A)** Normal control, **(B)** Leukemia control, **(C)** Normal + EOLE, **(D)** Leukemia + EOLE. In these figures, the brown spots indicate the intensity of BCLA1. The red arrow indicates the BCL2A1positive leukemic blast cells in the spleen, and the green arrow indicates the BCL2A1 negative spleen cells.

In progressive leukemia, the leukemic blast infiltrates into various bodily organs like the spleen. Therefore, the splenic infiltration of blast cells was investigated in all the groups using immunohistochemical staining (**Figure 9**). The immunohistochemistry of splenic sections revealed substantially lesser blast cell infiltration in the EOLE-treated leukemic mice compared to the leukemic control mice.

## 4 Discussion

The life-threatening side effects of existing therapeutics and the acquisition of drug resistance pushing the research globally that is targeted to develop new therapeutics for the treatment of leukemia which is less toxic as well as effective for the treatment of leukemia. The initial development of leukemia starts due to some genetic abnormalities catalysing signaling defects and abnormal cellular function. Chronic inflammation and apoptotic resistance are the two major abnormal functions that aid in the growth of cancer and spread to neighbouring tissues. The drugs that can alter inflammation and induce apoptosis in cancer cells help in preventing cancer growth. Plant phytochemicals are one of the interesting classes of molecules undergoing vigorous checking with the aim to identify novel anticancer molecules. Some noteworthy plant phytochemicals useful against leukemia are curcumin, lupeol, and corydine, isolated from certain herbs and trees (35). Another tree that has drawn the attention of researcher is the olive tree. The olive leaves, fruits, and oils contain many pharmacologically active phytochemicals like oleuropein and hydroxytyrosol (14). The GC-MS analysis of the prepared EOLE revealed the presence of oleuropein and hydroxytyrosol as major chemical structures (16). Both of these phytochemicals exerts anticancer activity through antioxidant, inflammatory, immunomodulatory mechanisms (14, 16) encouraged us to hypothesize and test the anti-leukemic activity of EOLE in an animal model of leukemia.

Before beginning with the *in-vivo* studies, the functional activity of EOLE was checked *in-vitro* on the K562 cell line. The EOLE induced apoptosis in K562 cells without any significant toxicity to the normal mouse splenocytes is consistent with the findings of previous studies carried out with olive leaf extracts on HL-60 and K562 cell line (14, 15). As a novel development to the existing literature, this study for the first time determined the effect of EOLE in the expression of apoptosis related proteins BCL-2, BCL-xL, BAX, PUMA and cytochrome c in the K562 cells. These proteins are categorized into pro-apoptotic and anti-apoptotic groups serve essential role in cellular apoptosis program (36). The EOLE was found to up-regulate the pro-apoptotic protein and trigger apoptosis in K562 cells. The results from the *in-vitro* studies sufficiently justify the plans to evaluate the activity of EOLE in leukemic mice.

The leukemia in mice is characterized by appearance of blast and very high WBC count in peripheral blood (24). The normal BM contains about 5% blast cells; however, the number of blast cells significantly increases in leukemic BM. This blast cell from the BM enters into blood stream and then infiltrates to other organs. The blast cell counts in the BM and peripheral blood are markers for the disease progression and the efficacy of any therapy (37). In the current study, the administration of EOLE in leukemic mice slow down the disease progression as indicated by significantly lesser WBC and blast count in the peripheral blood of EOLE-treated leukemic mice compared to the leukemic control. This data is consistent with the result obtained from the studies with standard anti-leukemic drug (38). Loss of body weight is an obvious feature of progressive disease (39). The body weight and serum LDH activity measured indicate better health of the leukemic mice that received EOLE treatment than the untreated mice. The high level of LDH in cancer is associated with inflammation, cell proliferation and survival, metastasis, and immune escape (40).

In order to explore the anti-leukemic mechanism of EOLE, various parameters in the BM that influence normal function of the BM were studied in the included group of mice. The abnormal expression of inflammatory cytokines and apoptosis regulatory proteins is a common phenomenon in leukemia and other hematological malignancies (10, 41, 42). The present study revealed elevated ROS, inflammatory cytokine, high NF-κB, ERK1/2, and high BCL2A1, MCL-1, and BCL-xL in the BM of leukemic mice. ROS is a critical mediator of inflammation and play important role in initiating malignant diseases (43). Elevated levels of ROS induce the expression of COX‐2 and cytokines like IL‐1β, TNF‐α, IL‐6, and IL‐8, promotes chronic inflammation (44, 45). The increased release of TNF-α and IL-1 in turn induces the expression of NF-κB which then stimulates the expression of inflammation-related genes like iNOS, Cox-2, several inflammatory cytokines and chemokines that contributes in abnormal cell proliferation, and alters normal apoptosis (8). By doing this, the NF-κB and inflammatory cytokine establish an intricate system for cancer growth that plays a seminal role in developing cancer (46). The NF-κB also directly regulates the expression of BCL2A1, an anti-apoptotic protein important for the survival of hematopoietic cells. Other factors like GM-CSF, TNF-α, and IL-1 up-regulates the expression of BCL2A1 protein in the hematopoietic compartment, leading to increased stem cell survival (10). It is evident from the previous studies that the hematopoietic stem and progenitor cells overexpress anti-apoptotic BCL2 family proteins: BCL2A1, BCL-xL become less susceptible or resistant to apoptotic stimulus and transformed into malignant cells (10, 11). Similarly, over-expression of MCL-1 in hematopoietic tissues sustains the growth and progression of AML and develops therapeutic resistance against various chemo-drugs (47). The excessive secretion of cytokine and growth factor caused by the elevated ROS and NF-κB activity leads to hyper-activation of ERK1/2 that influences cellular functions like proliferation and differentiation etc. (48, 49).

The excessive ROS bought up chronic inflammation. In opposite, neutralization of ROS inhibits carcinogenesis and related events (43) made ROS an important therapeutic target. The Oleuropein and Hydroxytyrosol present in EOLE are excellent ROS scavengers (50). In the present study, EOLE treatment to leukemic mice reduce the production of ROS, down-regulate NF-κB, and inflammatory markers like IL-1, TNF-α, iNOS, and COX-2 are consistent with the findings of previous studies (50). In the present study, the EOLE has reduced inflammation in the BM of leukemic mice and the release of TNF-α and IL-1β from LPS-induced inflamed mouse peritoneal macrophages consistently supports the anti-inflammatory activity of EOLE (50). The over-expression of NF-κB in the leukemic BM confers apoptotic resistance to the leukemic cells (8). The olive polyphenols indirectly down-regulates the expression of NF-κB help in alleviating inflammation and apoptotic resistance by regulating the expression of inflammatory cytokines and the proteins of apoptosis pathway. The role of NF-κB in leukemogenesis is evident from the studies where blocking the expression of NF-κB using an inhibitor molecule BMS-345541 altered the expression of genes central to leukemogenesis in patient-derived AML cells (51). The inhibition of NF-κB brings substantial changes in cytokine and interleukin signaling, cellular metabolism, and cell communication. In the present study, the EOLE-treatment significantly reduced the expression of NF-κB and altered the expression of BCL2A1, BCL-xL, and MCL-1 in the BM of leukemic mice. These are consistent with the available literature on olive polyphenols from previous studies (50). The NF-κB inhibitory and apoptosis-inducing functions of EOLE are also evident from the study of Liu and colleagues (52), where oleuropein, a major polyphenol in EOLE, induced apoptosis in breast cancer cells by abrogating the NF-κB activation cascade. Given the critical role of NF-κB in transcriptional control of a wide array of cellular genes involved in the initiation, maintenance, and progression of malignant diseases, including leukemia, it is not surprising that reducing its expression would significantly change cellular function. Therefore, the reduced inflammatory cytokines and anti-apoptotic proteins in the BM of EOLE-treated leukemic mice may have some association with down-regulated NF-κB in response to EOLE treatment. The reduced production of inflammatory cytokines and ROS further down-regulates the ERK1/2 activity stops cancer progression. It is evident from the studies in which therapeutic inhibition of ERK1/2 reduce the expression of MMPs and delays growth and invasion tumors, including acute myeloid leukemia (AML) (48, 53). The activity of EOLE delays the progression of leukemia and as a result lesser number of blast cell infiltrates in the spleen.

## 5 Conclusion

Overall, the present study investigated the anti-leukemic activity of EOLE *in-vitro* against K562 cell line and *in-vivo* against ENU-induced leukemia in BALB/c mice. The EOLE treatments to leukemic mice reduce ROS production and down-regulate the expression of NF-κB in the BM. Subsequently, the EOLE reduces the expression of several hematopoietic growth factors and inflammatory cytokines. The EOLE treatment also reverses the expression of apoptosis regulatory proteins in the BM of leukemic mice from an apoptosis-resistant presentation to apoptosis supporting one may indicate apoptosis induction in leukemic cells.

## Data availability statement

The original contributions presented in the study are included in the article/ **Supplementary material**. Further inquiries can be directed to the corresponding author.

## Ethics statement

The animal study was reviewed and approved by Institutional Animal Ethics Committee Tripura University.

## Author contributions

DM: Conceptualization, Validation, Resources, Supervision, Writing- Reviewing and Editing. PN: Methodology, Investigation, Formal analysis, Data Curation, Writing- Original Draft. SMo: Methodology, Data Curation. TA: Methodology, Data Curation. SMa: Methodology, Data Curation. AG: Methodology, Data Curation. RS: Methodology, Data Curation. MD: Material provided and review the manuscript. BS: Review manuscript and edit. All authors contributed to the article and approved the submitted version.
